# Prostate cancer survival according to socioeconomic and tumor characteristics in Manizales, Colombia

**DOI:** 10.17843/rpmesp.2025.424.14721

**Published:** 2025-12-12

**Authors:** Alexandra Giraldo-Osorio, Juan David Ladino, Miguel Ángel Giraldo Restrepo, Luisa Fernanda Vargas Dussan, Nelson Arias-Ortiz

**Affiliations:** 1 Health Promotion and Disease Prevention Research Group (GIPSPE), Manizales Population-Based Cancer Registry (RPCa-M), Department of Public Health, Universidad de Caldas, Manizales, Colombia. Universidad de Caldas Health Promotion and Disease Prevention Research Group (GIPSPE), Manizales Population-Based Cancer Registry (RPCa-M) Department of Public Health Universidad de Caldas Manizales Colombia; 2 Epidemiology Seedbed (EPICALDAS), Universidad de Caldas, Caldas, Colombia. Universidad de Caldas Epidemiology Seedbed (EPICALDAS) Universidad de Caldas Caldas Colombia

**Keywords:** cancer, prostate neoplasms, health inequities, epidemiology, survivorship

## Abstract

**Objective.:**

To estimate prostate cancer survival according to socioeconomic and tumor characteristics in the municipality of Manizales, Colombia, during the 2008-2018 period, based on population-based data.

**Materials and methods.:**

A population-based retrospective cohort study was conducted, including all incident cases of primary prostate cancer diagnosed in Manizales between 2008 and 2018, recorded in the Manizales Population-Based Cancer Registry. Overall survival was estimated using the Kaplan-Meier method, both for the total cohort and according to health insurance regimen, area of residence, socioeconomic position, age groups, histological type, essential Tumor-Node-Metastasis (TNM) classification, and risk according to Gleason score. The association between the variables of interest and survival was evaluated using Cox regression models.

**Results.:**

The overall five-year survival was 76.5%. Significant differences in survival were identified according to the health insurance regimen, with the risk of death before five years being approximately twice as high in patients from the subsidized and non-affiliated regimen compared to those from the contributory, special, and exception regimen.

**Conclusions.:**

Prostate cancer survival in Manizales is lower than that reported in populations with higher levels of development. Significant gaps in survival persist according to the health insurance regimen, disadvantaging the most socioeconomically vulnerable population, possibly mediated by late diagnoses due to barriers in timely access to treatment.

## INTRODUCTION

Prostate cancer (PCa) is the second highest incidence neoplasm in men, after lung cancer, and the fifth cause of cancer death worldwide as of 2022, according to the Global Cancer Observatory ^1^. Likewise, in 2023, in the United States, PCa represented 29% of all diagnosed cancers in men and ranked first in incidence ^1^^,^^2^. According to GLOBOCAN 2022 estimates ^3^, 16,479 new cases were registered in Colombia, corresponding to 29.3% of all new cancer cases in men. Furthermore, PCa survival is lower in older individuals ^4^ and also in developing countries, where the five-year survival rate is close to 80.3%, compared to developed countries, where it approaches 100% ^5^.

In Colombia, multidimensional poverty has been identified as a factor that delays early PCa diagnosis, which could contribute to increased mortality ^6^. National studies have demonstrated that age, Gleason score, prostate-specific antigen (PSA) levels, and the presence of metastasis influence five-year survival ^7^^,^^8^. A population-based study conducted in Manizales during the 2003-2007 period compared the survival of different types of cancer according to health insurance regimen and evidenced that patients belonging to the subsidized regimen or without affiliation presented less favorable survival than those enrolled in the contributory, special, and exception regimens (population with payment capacity) ^9^. Similarly, a study conducted in 2014 in Cali, Colombia, which analyzed the survival of patients diagnosed with PCa between 1995 and 2004, reported lower survival in patients of middle and low socioeconomic status compared to those of high status ^10^. In other countries, such as Mexico and the United States, PCa mortality has been related to both distance to urban centers and health services as well as higher poverty rates in the areas of residence of the cases ^11^^-^^13^.

To date, no updated population-based studies have been identified that evaluate PCa survival in Colombian populations and the possible associated socioeconomic inequities. Therefore, the objective of this study was to estimate prostate cancer survival according to socioeconomic and tumor characteristics in the municipality of Manizales, Colombia, based on population-based data.

**KEY MESSAGES**

Motivation for the study. Prostate cancer is the second highest incidence neoplasm in men worldwide and the fifth cause of cancer death as of 2022. In Colombia, it represented 29.3% of all new cancer cases in men during that year.

Main findings. Overall five-year survival was 76.5%. Significant gaps were identified according to the health insurance regimen, with a disadvantage for the most socioeconomically vulnerable population.

Implications. The observed survival is lower than that reported in countries with higher levels of development. Inequalities in survival could be mediated by late diagnoses and barriers to timely access to treatment.

## MATERIALS AND METHODS

### Design and study population

A population-based retrospective cohort study was conducted, consisting of all cases of primary prostate cancer diagnosed in the municipality of Manizales between 2008 and 2018, captured by the Manizales Population-Based Cancer Registry. The study area corresponds to Manizales, capital of the department of Caldas, a city with approximately 459,262 inhabitants, according to population projections from the National Administrative Department of Statistics (DANE, Spanish acronym) for 2025, of whom 93.8% reside in urban areas. Manizales is located at 2,150 meters above sea level in the Andean region of Colombia and has medium and high-complexity infrastructure for cancer diagnosis and treatment, including chemotherapy and radiotherapy services.

Cases were obtained from the Manizales Population-Based Cancer Registry (RPCa-M), which is part of the National Cancer Information System of Colombia (Infocáncer) ^14^. This registry is indexed with the International Agency for Research on Cancer and operates under its technical and methodological guidelines ^15^^,^^16^. The RPCa-M actively collects information on new cases of invasive malignant neoplasms diagnosed in residents of the municipality of Manizales, in both urban and rural areas. Data come from medical records, pathology reports, imaging, clinical laboratory, endoscopies, as well as national public health surveillance systems and vital statistics. Quality control protocols are periodically applied to the data using the IARC/IACR Cancer Registries Tools and Link Plus tools ^17^.

### Selection criteria

All incident cases with a diagnosis of primary prostate cancer between January 1, 2008, and December 31, 2018, according to the International Classification of Diseases for Oncology, third edition, first revision (topographic code C61.9), recorded in the RPCa-M database, were included. It should be noted that the registry only considers primary prostate tumors as incident, excluding metastatic tumors originating in other primary tissues. Cases identified solely by death certificate and those where it was confirmed that the patient did not reside in the jurisdiction of the municipality of Manizales at the time of diagnosis were excluded.

### Event definition and case follow-up

The event of interest was death from any cause, as the specific cause of death was not available. Passive follow-up was performed by consulting administrative databases of the Colombian Government, specifically the Unique Affiliate Database (BDUA, Spanish acronym) of the Administrator of the Resources of the General Social Security System in Health (ADRES, Spanish acronym) and the electoral census, using each patient’s identification number. In cases not located by passive follow-up, active follow-up was carried out through the review of medical records in oncology care centers. The index date corresponded to the diagnostic date recorded in the RPCa-M database, and the final follow-up date was considered as the last record in the BDUA or electoral census, the date of death, or the study closing date (July 31, 2024), whichever occurred first.

### Socioeconomic variables

Socioeconomic position (SEP) was determined by georeferencing the patient’s residence address at the time of diagnosis, using the Google Maps tool to identify the neighborhood (urban area) or district (rural area). Based on this information, cases were classified into low SEP (strata 1 and 2), middle SEP (strata 3 and 4), and high SEP (strata 5 and 6), according to the predominant socioeconomic stratum of the neighborhood or district according to the Housing-Household-Persons (VIHOPE) database of the National Administrative Department of Statistics (DANE). This classification corresponds to the guidelines of the DANE expert panel, which defines stratum 1 as low-low, 2 as low, 3 as middle-low, 4 as middle, 5 as middle-high, and 6 as high ^18^.

In Colombia, health coverage is mainly organized into two schemes: the subsidized regimen, aimed at the poor population or those without payment capacity, and the contributory, special, and exception regimens, for the population with payment capacity. The health insurance regimen (HIR) was determined from information available in the RPCa-M and consultation in the Unique Affiliate Database (BDUA), and was classified into five categories: i) special/exception, ii) contributory, iii) subsidized, iv) without affiliation, and v) without data. The area of residence (AR) was defined according to the location of the home at the time of diagnosis (urban or rural). Cases residing in rural areas (n = 40; 3.1%) were classified as belonging to low SEP, according to the predominant socioeconomic characteristics of the villages where they were registered.

### Patient and tumor variables

Age at diagnosis, topography, and morphology were obtained from the RPCa-M database and defined according to the International Classification of Diseases for Oncology, third edition, first revision (ICD-O 3.1). For cases with unknown age (n = 11), the median was imputed. The Gleason score and staging were obtained from histopathology reports and medical records. For staging, the essential Tumor, Node, Metastasis (TNM) system criteria proposed by the International Agency for Research on Cancer (IARC) for population-based studies were applied ^19^. The Gleason score was categorized as low risk (Gleason < 7), intermediate risk (Gleason = 7), and high risk (Gleason > 7), according to the D’Amico classification ^20^^,^^21^.

### Statistical analysis

The variables included were qualitative in nature, measured on nominal or ordinal scales, and described using absolute and relative frequencies. All incident cases (N = 1,275) were included in the descriptive analysis; for the survival analysis, cases captured solely by death certificate (DCO) were excluded, because their follow-up time is 0 days and they tend to underestimate survival ^22^.

Survival time was calculated as the difference between the date of incidence and the date of last contact, death, loss to follow-up, or study closure. Survival at 1, 3, and 5 years was estimated using the Kaplan-Meier method for the total cohort and according to categories of HIR, AR, SEP, age groups (< 50 years, 51-69 years, and ≥ 70 years), histology (adenocarcinoma vs. others), essential TNM, and risk according to Gleason score (low, intermediate, and high). Differences between survival functions were evaluated using the log-rank test.

Univariate and multivariate analyses were performed using Cox proportional hazards models. Model A included HIR or SEP (analyzed separately), adjusted for age, histology, essential staging, and Gleason risk classification. Model B simultaneously included HIR and SEP, in addition to adjustment variables. The proportional hazards assumption was verified using Schoenfeld residuals. Interaction terms between insurance and staging, as well as between SEP and staging, were evaluated. Due to the low number of observations in some categories, HIR was regrouped into two categories: i) contributory + special + exception (population with payment capacity) and ii) subsidized + without affiliation (poor population or without payment capacity). Age was recoded into two categories with a cutoff point at 70 years. In the multivariate models, “no data” or “unknown” categories and DCO cases were excluded. The outcome was overall 5-year survival, considering death from any cause.

**Figure 1:**
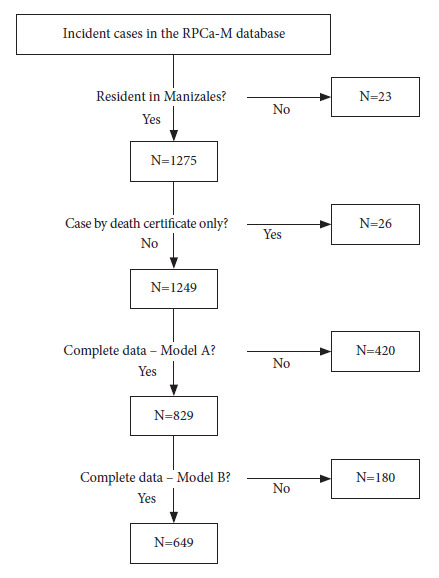
Flowchart of the selection of incident prostate cancer cases (2008-2018) in the Population-based Cancer Registry of Manizales.

Statistical analysis was performed using Stata version 16.1. Figure 1 presents the case selection flow chart.

### Ethical considerations

The research was approved by the Bioethics Committee of the Universidad de Caldas through Minutes No. 003 of June 24, 2024. It was classified as a minimum-risk study and conformed to the provisions of Resolution 8430 of 1993 of the Ministry of Health of Colombia ^23^.

## RESULTS

**Figure 2:**
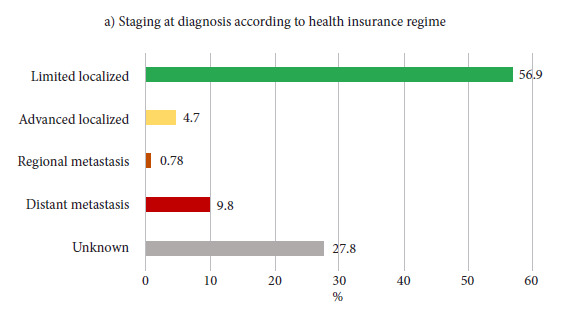
Coverage and frequency of the essential Tumor-Node-Metastasis variable in prostate cancer cases. Manizales, 2008-2018.

**Figure 3:**
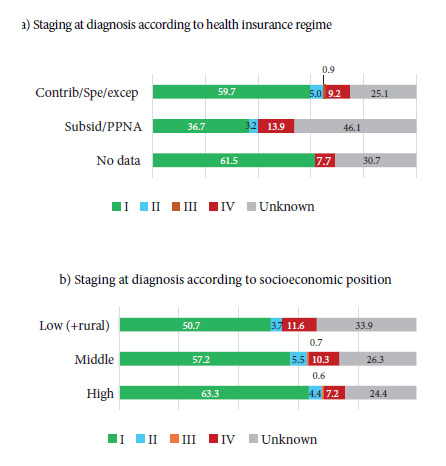
Distribution of essential Tumor-Node-Metastasis staging according to a) health insurance regime and b) socioeconomic position

In Manizales, 1,275 incident cases of PCa were registered during the study period. The median age at diagnosis was 70 years and the average was 69.8 years (SD: 9.6). Regarding the health insurance regimen (HIR), 85.1% of patients belonged to the contributory regimen. The predominant socioeconomic position (SEP) was middle, with 47.0% of cases. 76.3% resided in the urban area at the time of diagnosis. 26 DCO cases (2.0%) were identified. Socio-demographic and tumor characteristics are presented in Table 1. The morphological codes registered in the RPCa-M were 8000, 8010, 8140, 8500, 8550, 8574, and 8801, all with malignant behavior. Figure 2 shows the distribution by staging at diagnosis. Staging according to HIR and SEP is presented in Figure 3, evidencing a significant association between HIR and essential TNM staging, with a higher proportion of diagnoses in limited localized stage among those affiliated with the contributory, special, and exception regimens, compared to those affiliated with the subsidized regimen and the non-affiliated (59.7% vs. 36.7%; p < 0.0001). Annex 1 presents the distribution of missing data according to event status.

**Table 1:** Sociodemographic and clinical characteristics of patients diagnosed with prostate cancer, according to event status. Manizales, 2008-2018.

		Alive n (%)	Deceased n (%)	Lost to follow-up n (%)	p-value*
All cases		719 (56.4)	552 (43.3)	4 (0.3)	
Age					
	< 50 years	17 (2.4)	2 (0.4)	1 (25.0)	p<0.0001
	50 a 69 years	460 (64.0)	149 (27.0)	1 (25.0)	
	70 years and older	242 (33.6)	401 (72.6)	2 (50.0)	
Health Insurance Regimen					
	Special/exception	29 (4.0)	17 (3.1)	0 (0.0)	p<0.0001
	Contributory	630 (87.6)	425 (77.0)	3 (75.0)	
	Subsidized	48 (6.7)	105 (19.0)	0 (0.0)	
	Without affiliation	4 (0.6)	1 (0.2)	0 (0.0)	
	No data	8 (1.1)	4 (0.7)	1 (25.0)	
Socioeconomic Position					
	High (strata 5 and 6)	117 (16.3)	62 (11.2)	1 (25.0)	p=0.050
	Middle (strata 3 and 4)	333 (46.3)	265 (48.0)	1 (25.0)	
	Low (strata 1, 2, and rural)	111 (15.4)	105 (19.0)	0 (0.0)	
	No data	158 (22.0)	120 (21.8)	2 (50.0)	
Area of Residence					
	Urban	538 (74.8)	418 (75.7)	3 (75.0)	p=0.920
	Rural	24 (3.3)	15 (2.7)	0 (0.0)	
	No data	157 (21.9)	119 (21.6)	1 (25.0)	
Histology					
	Adenocarcinoma	652 (90.7)	386 (69.9)	4 (100.0)	p<0.0001
	Others** and non-specified	67 (9.3)	166 (30.1)	0 (0.0)	
Essential TNM					
	Limited localized	486 (67.6)	236 (42.8)	3 (75.0)	p<0.0001
	Advanced localized	47 (6.5)	13 (2.4)	0 (0.0)	
	Regional metastasis	9 (1.3)	1 (0.1)	0 (0.0)	
	Distant metastasis	37 (5.1)	88 (15.9)	0 (0.0)	
	Unknown	140 (19.5)	214 (38.8)	1 (25.0)	
Risk according to Gleason					
	High	114 (15.9)	111 (20.1)	2 (50.0)	p<0.0001
	Intermediate	209 (29.1)	143 (25.9)	1 (25.0)	
	Low	325 (45.2)	116 (21.0)	1 (25.0)	
	No data	71 (9.8)	182 (33.0)	0 (0.0)	
	Diagnosis period				
	2008-2012	203 (28.2)	268 (48.6)	1 (25.0)	p<0.0001
	2013-2018	516 (71.8)	284 (51.4)	3 (75.0)	

* Fisher’s exact test, excluding the “no data” category.

** Spindle cell sarcoma and malignant neoplasm.

Stage according to health insurance: p<0.0001. Stage according to socioeconomic position: p=0.16.

**Figure 4:**
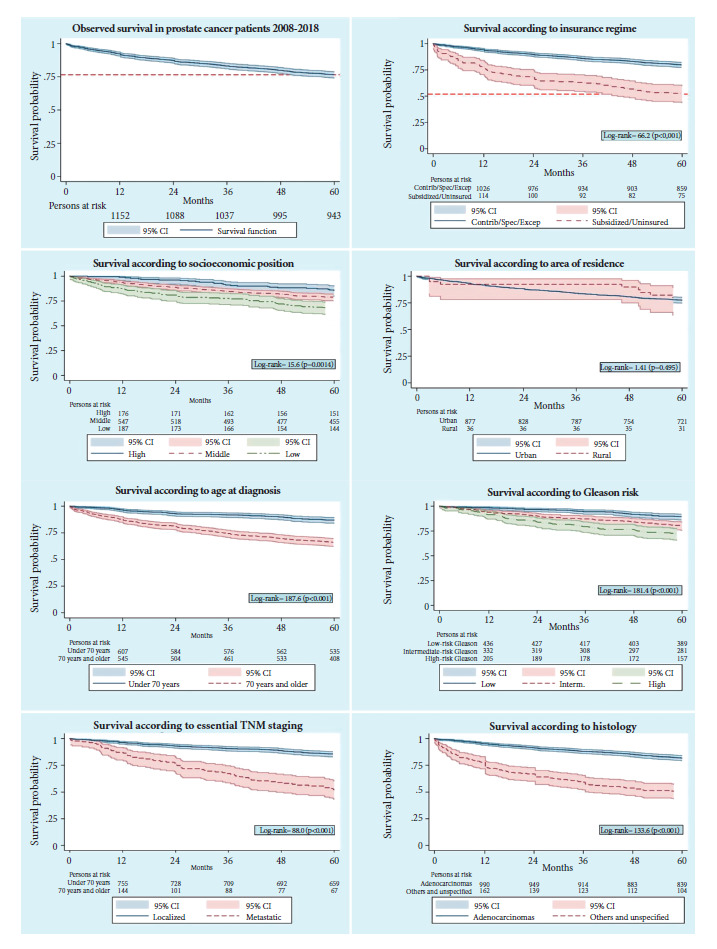
Kaplan-Meier functions according to study variables.

The overall five-year survival observed for the total cohort was 76.5%. Figure 4 presents the Kaplan-Meier curves according to HIR, SEP, Gleason score, and essential TNM. Table 2 shows the overall survival (OS) estimates at 1, 3, and 5 years per variable. Significant differences in 5-year OS were observed according to age, with lower survival in those over 70 years. According to HIR, the 5-year OS of patients affiliated with the subsidized regimen was 32 and 27 percentage points lower than that of those affiliated with the special/exception and contributory regimens, respectively. Regarding SEP, cases classified as low SEP presented a 5-year OS approximately 11 and 18 percentage points lower than that observed in middle and high SEP, respectively. No significant differences in OS were identified according to the area of residence. Histology was significantly associated with OS, being 30 percentage points higher in patients with adenocarcinoma compared to other non-specified histological types (81.7% vs. 50.5%). Stage IV patients presented a five-year OS between 35 and 40 percentage points lower than those in early stages. According to Gleason risk, high-risk patients had a five-year OS 17 percentage points lower than low-risk patients. No significant differences were observed by diagnostic period.

**Table 2:** Overall prostate cancer survival according to sociodemographic and tumor characteristics. Manizales, 2008-2018.

		N*	Deaths	Overall survival			
				12 months (95% CI)	36 months (95% CI)	60 months (95% CI)	Log-rank**
All cases		1249	526	92.3 (90.1-93.7)	83.2% (81.0-85.1)	76.5% (74.0-78.8)	
Age							
	< 50 years	20	2	100% (-)	94.7% (68.1-99.2)	89.5% (64.1-97.3)	p<0.001
	50 a 69 years	609	148	96.6% (94.8-97.7)	91.8% (89.3-93.7)	86.8% (83.9-89.3)	
	70 years and older	620	376	87.9% (85.1-90.2)	74.4% (70.7-77.6)	66.0% (62.1-69.6)	
Health insurance							
	Special/exception	45	16	97.8 (85.3-99.7)	91.1 (78.0-96.6)	84.4 (70.1-92.3)	p<0.001
	Contributory	1040	407	93.9% (92.3-95.2)	85.5 (83.2-87.5)	79.4 (76.8-81.7)	
	Subsidized	146	98	78.1 (70.5-84.0)	63.0 (54.6-70.3)	52.0 (43.6-59.7)	
	Without affiliation	5	1	100 (-)	100 (-)	100 (-)	
	No data	13	4	100 (-)	91.7 (53.9-98.8)	83.3 (48.2-95.6)	
Socioeconomic position							
	High (strata 5 and 6)	178	60	98.9 (95.6-99.7)	91.0 (85.8-94.4)	85.4 (79.3-89.8)	p=0.001
	Middle (strata 3 and 4)	584	250	93.7 (91.4-95.4)	84.4 (81.2-87.1)	78.6 (75.0-81.7)	
	Low (strata 1, 2, and rural)	214	103	87.4 (82.1-91.2)	77.6 (71.4-82.6)	67.7 (61.0-73.6)	
	No data	273	113	89.0 (84.6-92.2)	79.8 (74.5-84.1)	73.1 (67.4-77.9)	
Area of residence							
	Urban	940	399	93.3 (91.5-94.7)	83.8 (81.3-86.0)	77.4 (74.6-80.0)	p=0.495
	Rural	39	15	92.3 (78.0-97.5)	92.3 (78.0-97.5)	79.5 (63.1-89.2)	
	No data	270	112	88.9 (84.4-92.1)	79.6 (74.2-83.9)	72.8 (67.1-77.7)	
Histology							
	Adenocarcinoma	1042	386	95.2 (93.7-96.3)	88.0 (85.8-89.8)	81.7 (79.2-83.9)	p<0.001
	Others and non-specified	207	140	77.9 (71.6-82.9)	59.1 (52.1-65.5)	50.5 (43.5-57.0)	
Essential TNM							
	Limited localized	725	236	96.1 (94.4-97.3)	90.3 (87.9-92.3)	85.2 (82.4-87.6)	p<0.001
	Advanced localized	60	13	98.3 (88.6-99.8)	93.3 (83.2-97.4)	88.3 (77.1-94.3)	
	Regional metastasis	10	1	100 (-)	90.0 (47.3-98.5)	90.0 (47.3-98.5)	
	Distant metastasis	121	84	86.0 (78.4-91.0)	65.3 (56.1-73.0)	48.7 (39.5-57.2)	
	No data	333	192	85.0 (80.7-88.4)	72.1 (66.9-76.6)	65.2 (59.8-70.0)	
Risk according to Gleason							
	High	226	110	91.5 (87.0-94.5)	79.5 (73.6-84.2)	71.8 (75.4-77.2)	p<0.001
	Intermediate	353	143	94.1 (91.0-96.1)	87.3 (83.3-90.3)	80.2 (75.6-84.0)	
	Low	442	116	98.6 (97.0-99.4)	94.6 (92.0-96.3)	89.3 (86.1-91.9)	
	No data	228	157	78.2 (72.2-83.0)	58.5 (51.9-64.6)	50.7 (44.0-56.9)	
Diagnosis period							
	2008-2012	458	254	92.6 (89.8-94.7)	83.0 (79.3-86.2)	76.7 (72.5-80.3)	>p=0.839
	2013-2018	791	272	92.2 (90.0-93.8)	83.3 (80.5-85.7)	76.4 (73.2-79.2)	

* Excludes 26 cases identified by death certificate.

** Excluding “no data” categories.

Table 3 presents the results of the multivariate analysis. A significant effect of HIR on 5-year OS was evidenced: patients affiliated with the subsidized regimen and those without affiliation —the most socially vulnerable population— presented twice the risk of dying before five years from diagnosis, compared to patients with payment capacity affiliated with the contributory, special, or exception regimens, regardless of socioeconomic position, age, histology, staging, and risk according to Gleason score. The effects observed for SEP in the bivariate analysis lost statistical significance in the multivariate model after adjustment for the other covariates.

**Table 3:** Cox proportional hazards models for prostate cancer survival according to health insurance and socioeconomic position. Manizales, 2008-2018.

		Univariate analysis	Multivariate analysis	
		HR (95% CI)	Model A	Model B
			HR (95% CI)	HR (95% CI)
Insurance				
	Contributory + Special/Excep.	Ref.	Ref.	Ref.
	Subsidized + Without affiliation	2.67 (2.14 - 3.33)	2.23 (1.60 - 3.11)	2.58 (1.77 - 3.77)
Socioeconomic level				
	High	Ref.	Ref.	Ref.
	Middle	1.38 (1.04 - 1.83)	1.42 (0.97 - 2.07)	1,30 (0.89 - 1.90)
	Low	1.85 (1.35 - 2.55)	1.91 (1.22 - 2.96)	1.47 (0.93 - 2.32)
Age				
	<70 years	Ref.	Ref.	Ref.
	70 years and older	3.48 (2.87 - 4.21)	2.87 (2.24 - 3.68)	2.65 (2.01 - 3.50)
Histological subtype				
	Adenocarcinomas	Ref.	Ref.	Ref.
	Others and non-specified*	2.96 (2.44 - 3.60)	1.26 (0.71 - 2.20)	1.56 (0.86 - 2.83)
Stage (Essential TNM)				
	I - Limited localized	Ref.	Ref.	Ref.
	II - Advanced localized	0.68 (0.39 - 1.19)	0.69 (0.36 - 1.31)	0.88 (0.46 - 1.68)
	III - Regional metastasis	0.23 (0.03 - 1.65)	0.25 (0.03 - 1.77)	0.47 (0.07 - 3.38)
	IV - Distant metastasis	3.60 (2.80 - 4.64)	2.19 (1.61 - 2.98)	2.15 (1.51 - 3.06)
Gleason risk classification				
	I - Low risk	Ref.	Ref.	Ref.
	II - Medium risk	1.66 (1.30 - 2.12)	1.59 (1.19 - 2.12)	1.34 (0.98 - 1.85)
	III - High risk	2.41 (1.85 - 3.12)	2.35 (1.73 - 3.19)	2.09 (1.48 - 2.95)

* Others and non-specified included one case of spindle cell sarcoma and 232 cases with non-specified histology. Model A includes insurance or socioeconomic position (one at a time) adjusted for the other variables. Model A for insurance was adjusted based on 829 observations and yielded a Likelihood ratio (LR) = 182.68; Model A for socioeconomic level was adjusted based on 652 observations and yielded an LR = 116.84. Model B was adjusted with n = 649 (number of observations with known data for all variables included in the model) and includes insurance and socioeconomic position (simultaneously), in addition to the adjustment variables; Model B yielded an LR = 136.13. The proportionality assumption was met, except for the categories “Subsidized insurance” and “Low SEP” in Model A.

## DISCUSSION

This study updates the population-based survival estimates in Manizales and evaluates the effect of socioeconomic, patient, and tumor variables on the probability of five-year survival after PCa diagnosis. Compared to populations in developed countries, the survival observed in Manizales is lower than the five-year relative survival (RS) of 97.6% reported by Siegel in the United States ^24^; however, it should be noted that overall survival (OS) and RS estimates are not strictly comparable due to methodological differences. Survival in Manizales is also lower than the 81.2% reported in New Zealand by Matti and Zargar-Shoshtari ^25^, and the 89% reported by Barceló-Obrador using data from the Mallorca Cancer Registry, Spain ^26^. In the Latin American context, in Mexico, Torres-Sánchez *et al.*, for the 2012-2016 period, reported a 5-year OS of 62.0% in patients affiliated with the Seguro Popular ^27^, while, in Veracruz, Gutiérrez-Juárez et al. found an OS of 47.7% for the 2013-2017 period in a regional referral hospital ^11^.

In Colombia, survival in Manizales is lower than that reported in Cali, but higher than that registered in Bogotá. Bravo et al. analyzed the 1998-2017 period in the Cali Population-Based Cancer Registry and documented a net survival (NS) that varied according to the period: 79.9% (1998-2002), 90.2% (2003-2007), 87.5% (2008-2012), and 90.1% (2013-2017) ^28^. Although these estimates correspond to NS and not OS, they offer a national frame of reference. In contrast, in a hospital study in Bogotá, Campos-Guzmán reported a 5-year OS of 57.0% for the 2008-2014 period ^7^. Compared with the previous study in Manizales, the 5-year OS observed in this cohort shows a five percentage point improvement over the 71.1% reported by Arias-Ortiz and De Vries for 2003-2007 ^9^.

As expected, age over 70 years was associated with an approximately twice higher risk of dying before five years after diagnosis. According to the IARC, the incidence of PCa in people over 65 years increased steadily between 1988 and 2007, and it is projected that by 2030, developing countries will have a higher age-adjusted incidence than developed countries ^29^. The higher risk of death in older patients is consistent with that described by Bernard et al. in the United States, who reported that diagnosis at age 75 or older constitutes a significant risk factor for PCa mortality (HR: 1.5; 95% CI: 1.4-1.6) ^4^, as well as with previous hospital studies in Colombia ^7^^,^^8^.

Regarding socioeconomic position (SEP), the bivariate analysis showed a direct association with survival, evidencing lower OS as SEP decreases. In the US population, a higher risk of PCa death has been demonstrated in men with lower educational levels and who reside in lower-income neighborhoods, even in those with high educational levels but who live in disadvantaged environments (HR: 1.4; 95% CI: 1.1-1.8) ^30^. Similarly, the Swedish National Cancer Registry found that patients with lower socioeconomic status were diagnosed at more advanced stages, and that all-cause mortality was significantly higher in the lowest income quartile compared to the highest income quartile (30% vs. 12%), with higher cure rates in the most favored groups (OR: 1.8; 95% CI: 1.6-1.9) ^31^. In Mexico, Gutiérrez-Juárez *et al.* reported that the higher the degree of marginalization, the higher the probability of dying before five years (HR: 2.3; 95% CI: 1.5-3.7) ^11^. In Costa Rica, despite having a universal health system, social inequities in cancer survival have also been documented ^32^. In Colombia, Restrepo et al. found higher survival in people with middle and high SEP compared to low SEP in Cali ^10^.

Regarding the health insurance regimen (HIR), this study confirms that belonging to the subsidized regimen or being uninsured is an independent predictor of lower survival compared to being affiliated with the contributory, special, or exception regimens. In the United States, Krimphove *et al.* found that African American patients with advanced PCa present a higher risk of mortality associated, among other factors, with lower private insurance coverage ^33^. Similarly, Myint *et al.* reported significant differences in five-year survival between uninsured patients or those with public insurance versus patients with private insurance (HR between 1.3 and 2.1) ^34^. It has also been documented that public insurance or the absence of insurance is associated with less access to PCa treatment ^35^. In Manizales, survival differences according to HIR could be explained, at least partially, by barriers in access to early diagnosis, given that the proportion of initial stages was lower among subsidized regimen and non-affiliated patients ^9^. Although Colombian legislation guarantees oncology care regardless of the type of insurance ^36^, the results show that inequality gaps persist and require more effective interventions.

No significant differences in survival were found according to area of residence, in agreement with the previous study in Manizales ^9^. This finding contrasts with what was described in Veracruz, Mexico, where rural patients presented more advanced stages at diagnosis and lower survival (HR: 1.7; 95% CI: 1.2-2.4) ^11^. In Manizales, this result could be explained by the low proportion of rural population (6.3%), which limits the statistical power to detect differences ^37^.

Regarding tumor characteristics, the frequency of limited localized stage (59.6%) was lower than the sum of stages I and II reported in the national review by Solano-Dazzarola *et al.*^38^. The survival differences by staging observed were consistent with those described in the literature ^38^. Likewise, the results according to the Gleason score agree with hospital studies in Colombia ^7^^,^^8^ and with research from Mexico ^27^ and Spain ^26^^,^^39^. The Cox models used were based on the theoretical framework of health inequities and on previous evidence regarding the effect of socioeconomic position, rurality, and health insurance on oncology survival ^35^^,^^40^^-^^44^.

Among the main limitations of the study is the percentage of missing data in histology, staging, and Gleason score, which could have introduced information bias, particularly due to the higher proportion of missing data among the deceased. Additionally, the use of the neighborhood’s socioeconomic stratum as a proxy variable for individual SEP could generate residual confounding. Another relevant limitation is the lack of information on treatments received. Likewise, as specific causes of death were not available, only observed survival was estimated, which limits causal interpretation; in future studies, it would be desirable to apply net survival methods ^45^. Information on ethnicity, educational level, and therapeutic adherence, relevant variables in PCa survival, was also unavailable.

As strengths, this is a population-based study with ten years of follow-up, supported by a registry that meets international standards, which favors the comparability of the results. Its population nature allows it to reflect real health care conditions. The low percentage of losses to follow-up (<0.5%) strengthens the validity of the estimates.

In future research, it will be key to incorporate additional variables such as PSA levels, individual indicators of socioeconomic level, detailed treatment data, therapeutic adherence, and specific causes of death. Furthermore, studies with a qualitative approach will allow for a deeper understanding of the perception of the disease, access to care, and quality of life.

In conclusion, overall PCa survival in Manizales during the 2008-2018 period remains lower than that observed in developed countries. Approximately seven to eight out of ten patients survive five years after diagnosis. Significant survival gaps persist according to the health insurance regimen, disadvantaging the socially vulnerable population, possibly mediated by late diagnoses and barriers to treatment access, independent of socioeconomic position, age, histology, staging, and risk according to Gleason score.
