# Supplementary material for: Prostate cancer survival according to socioeconomic and tumor characteristics in Manizales, Colombia
Source: Rev Peru Med Exp Salud Publica. 2025 Dec 12;42(4):349–60. doi: 10.17843/rpmesp.2025.424.14721 (PMC12879986; doi:10.17843/rpmesp.2025.424.14721)
Supplement: Supplementary material. — Available in the electronic version of the RPMESP. [file rpmesp-42-04-14721-s001.docx]

**Material suplementario.**

| **Anexo 1. Número de casos (%) con valores perdidos para las variables del estudio según estatus del evento** | | | | |
| --- | --- | --- | --- | --- |
|  | **Vivo**  **(n=719)** | **Fallecido (n=552)** | **z** | **Valor p** |
| Nivel socioeconómico | 158 (21,97%) | 120 (21,74%) | 0,138 | 0,890 |
| Régimen de aseguramiento en salud | 8 (1,11%) | 4 (0,72%) | 0,742 | 0,458 |
| Zona de residencia | 157 (21,84%) | 119 (21,59%) | 0,132 | 0,895 |
| Edad | 10 (1,39%) | 1 (0,18%) | 3,268 | **0,001** |
| Histología no especificada | 67 (9,32%) | 166 (30,07%) | -9,48 | **<0,001** |
| TNM esencial | 140 (19,47%) | 214 (38,77%) | -7,91 | **<0,001** |
| Riesgo según Score de Gleason | 71 (9,87%) | 182 (32,97%) | -9,76 | **<0,001** |
