# Supplementary material for: Prostate cancer survival according to socioeconomic and tumor characteristics in Manizales, Colombia
Source: Rev Peru Med Exp Salud Publica. 2025 Dec 12;42(4):349–60. doi: 10.17843/rpmesp.2025.424.14721 (PMC12879986; doi:10.17843/rpmesp.2025.424.14721)
Supplement: Translation

# Translation: Sobrevida al cáncer de próstata según características socioeconómicas y del tumor en Manizales, Colombia

**Authors:** Alexandra Giraldo-Osorio, Juan David Ladino, Miguel Ángel Giraldo Restrepo, Luisa Fernanda Vargas Dussan, Nelson Arias-Ortiz

**Affiliations:** 1 Grupo de Investigación en Promoción de la Salud y Prevención de la Enfermedad (GIPSPE), Registro Poblacional de Cáncer de Manizales (RPCa-M), Departamento de Salud Pública, Universidad de Caldas, Manizales, Colombia.; 2 Semillero de Epidemiología (EPICALDAS), Universidad de Caldas, Caldas, Colombia.

**Keywords:** cáncer, neoplasias de la próstata, inequidades en salud, epidemiología, supervivencia

## Abstract

**Objetivo.:**

Estimar la sobrevida del cáncer de próstata según características socioeconómicas y del tumor en el municipio de Manizales, Colombia, durante el periodo 2008-2018, a partir de datos de base poblacional.

**Materiales y métodos.:**

Se realizó un estudio de cohorte retrospectiva de base poblacional que incluyó todos los casos incidentes de cáncer de próstata primario diagnosticados en Manizales entre 2008 y 2018, registrados en el Registro Poblacional de Cáncer de Manizales. La sobrevida global se estimó mediante el método de Kaplan-Meier, tanto para la cohorte total como según régimen de aseguramiento en salud, zona de residencia, posición socioeconómica, grupos etarios, tipo histológico, clasificación Tumor-Nódulo-Metástasis (TNM) esencial y riesgo según puntaje de Gleason. La asociación entre las variables de interés y la sobrevida se evaluó mediante modelos de regresión de Cox.

**Resultados.:**

La sobrevida global a cinco años fue de 76,5%. Se identificaron diferencias significativas en la sobrevida según el régimen de aseguramiento en salud, observándose un riesgo de muerte antes de los cinco años aproximadamente dos veces mayor en los pacientes del régimen subsidiado y no afiliados, en comparación con los del régimen contributivo, especial y de excepción.

**Conclusiones.:**

La sobrevida por cáncer de próstata en Manizales es inferior a la reportada en poblaciones con mayor nivel de desarrollo. Persisten brechas importantes en la sobrevida según el régimen de aseguramiento en salud, en desventaja de la población socioeconómicamente más vulnerable, posiblemente mediadas por diagnósticos tardíos debido a barreras en el acceso oportuno al tratamiento.

## INTRODUCCIÓN

El cáncer de próstata (CaP) es la segunda neoplasia de mayor incidencia en hombres, después del cáncer de pulmón, y la quinta causa de muerte por cáncer a nivel mundial para 2022, según el Observatorio Global del Cáncer ^1^. Asimismo, en 2023, en Estados Unidos, el CaP representó el 29% de todos los cánceres diagnosticados en hombres y ocupó el primer lugar en incidencia ^1^^,^^2^. De acuerdo con estimaciones de GLOBOCAN 2022 ^3^, en Colombia se registraron 16 479 casos nuevos, correspondientes al 29,3% de todos los casos nuevos de cáncer en hombres. Además, la sobrevida al CaP es menor en individuos de mayor edad ^4^^)^ y también en países en vías de desarrollo, donde la tasa de sobrevida a cinco años es cercana al 80,3%, en comparación con países desarrollados, donde se aproxima al 100% ^5^.

En Colombia, la pobreza multidimensional ha sido identificada como un factor que retrasa el diagnóstico temprano del CaP, lo cual podría contribuir al aumento de su mortalidad ^6^. Estudios nacionales han demostrado que la edad, el puntaje de Gleason, el nivel de antígeno prostático específico (PSA) y la presencia de metástasis influyen en la sobrevida a cinco años ^7^^,^^8^. Un estudio de base poblacional realizado en Manizales durante el período 2003-2007 comparó la sobrevida de diferentes tipos de cáncer según el régimen de aseguramiento en salud y evidenció que los pacientes pertenecientes al régimen subsidiado o sin afiliación presentaban una sobrevida menos favorable que aquellos adscritos a los regímenes contributivo, especial y de excepción (población con capacidad de pago) ^9^. De manera similar, un estudio realizado en 2014 en Cali, Colombia, que analizó la sobrevida de pacientes diagnosticados con CaP entre 1995 y 2004, reportó menor sobrevida en pacientes de estrato socioeconómico medio y bajo en comparación con los de estrato alto ^10^. En otros países, como México y Estados Unidos, la mortalidad por CaP se ha relacionado tanto con la distancia a los centros urbanos y a los servicios de salud como con mayores tasas de pobreza en las áreas de residencia de los casos ^11^^-^^13^.

Hasta el momento, no se han identificado estudios de base poblacional actualizados que evalúen la sobrevida al CaP en poblaciones colombianas y las posibles inequidades socioeconómicas asociadas. Por ello, el objetivo de este estudio fue estimar la sobrevida por cáncer de próstata según características socioeconómicas y del tumor en el municipio de Manizales, Colombia, a partir de datos de base poblacional.

**MENSAJE CLAVE**

Motivación para el estudio. El cáncer de próstata es la segunda neoplasia de mayor incidencia en hombres a nivel mundial y la quinta causa de muerte por cáncer para 2022. En Colombia, representó el 29,3% de todos los nuevos casos de cáncer en hombres durante ese año.

Principales hallazgos. La sobrevida global a cinco años fue del 76,5%. Se identificaron brechas significativas según el régimen de aseguramiento en salud, con desventaja para la población socioeconómicamente más vulnerable.

Implicaciones. La sobrevida observada es inferior a la reportada en países con mayor nivel de desarrollo. Las desigualdades en la sobrevida podrían estar mediadas por diagnósticos tardíos y barreras en el acceso oportuno al tratamiento.

## MATERIALES Y MÉTODOS

### Diseño y población de estudio

Se realizó un estudio de cohorte retrospectiva de base poblacional conformado por todos los casos de cáncer de próstata primario diagnosticados en el municipio de Manizales entre 2008 y 2018, captados por el Registro Poblacional de Cáncer de Manizales. El área de estudio corresponde a Manizales, capital del departamento de Caldas, ciudad que cuenta con aproximadamente 459 262 habitantes, según las proyecciones poblacionales del Departamento Administrativo Nacional de Estadística (DANE) para 2025, de los cuales el 93,8% reside en zona urbana. Manizales se localiza a 2 150 metros sobre el nivel del mar, en la región Andina de Colombia, y dispone de infraestructura de mediana y alta complejidad para el diagnóstico y tratamiento del cáncer, incluyendo servicios de quimioterapia y radioterapia.

Los casos se obtuvieron del Registro Poblacional de Cáncer de Manizales (RPCa-M), que hace parte del Sistema Nacional de Información en Cáncer de Colombia (Infocáncer) ^14^. Este registro se encuentra indexado ante la Agencia Internacional para la Investigación del Cáncer y opera bajo sus lineamientos técnicos y metodológicos ^15^^,^^16^. El RPCa-M recolecta de forma activa información de los casos nuevos de neoplasias malignas invasivas diagnosticadas en residentes del municipio de Manizales, tanto de la zona urbana como rural. Los datos provienen de historias clínicas, reportes de anatomía patológica, imágenes, laboratorio clínico, endoscopias, así como de los sistemas nacionales de vigilancia en salud pública y de estadísticas vitales. De manera periódica, se aplican protocolos de control de calidad de los datos mediante las herramientas IARC/IACR Cancer Registries Tools y Link Plus ^17^.

### Criterios de selección

Se incluyeron todos los casos incidentes con diagnóstico de cáncer de próstata primario entre el 1 de enero de 2008 y el 31 de diciembre de 2018, de acuerdo con la Clasificación Internacional de Enfermedades para Oncología, tercera edición, primera revisión (código topográfico C61.9), registrados en la base de datos del RPCa-M. Cabe precisar que el registro considera como incidentes únicamente los tumores primarios de próstata, excluyéndose los tumores metastásicos originados en otros tejidos primarios. Se excluyeron los casos identificados únicamente por certificado de defunción y aquellos en los que se constató que el paciente no residía en la jurisdicción del municipio de Manizales al momento del diagnóstico.

### Definición de evento y seguimiento de los casos

El evento de interés fue la muerte por cualquier causa, dado que no fue posible disponer de la causa específica de defunción. Se realizó un seguimiento pasivo mediante consulta en bases de datos administrativas del Gobierno de Colombia, específicamente la Base de Datos Única de Afiliados (BDUA) de la Administradora de los Recursos del Sistema General de Seguridad Social en Salud (ADRES) y el censo electoral, utilizando el número de identificación de cada paciente. En los casos no localizados por seguimiento pasivo, se efectuó seguimiento activo a través de la revisión de historias clínicas en los centros de atención oncológica. La fecha índice correspondió a la fecha de diagnóstico registrada en la base de datos del RPCa-M, y como fecha final del seguimiento se consideró la del último registro en la BDUA o en el censo electoral, la fecha de defunción o la fecha de cierre del estudio (31 de julio de 2024), lo que ocurriera primero.

### Variables socioeconómicas

La posición socioeconómica (PSE) se determinó mediante la georreferenciación de la dirección de residencia del caso al momento del diagnóstico, utilizando la herramienta Google Maps para identificar el barrio (área urbana) o el corregimiento (área rural). Con base en esta información, los casos se clasificaron en PSE baja (estratos 1 y 2), media (estratos 3 y 4) y alta (estratos 5 y 6), según el estrato socioeconómico predominante del barrio o corregimiento de acuerdo con la base de datos de Viviendas-Hogares-Personas (VIHOPE) del Departamento Administrativo Nacional de Estadística (DANE). Esta clasificación corresponde a los lineamientos de la mesa de expertos del DANE, que define el estrato 1 como bajo-bajo, el 2 como bajo, el 3 como medio-bajo, el 4 como medio, el 5 como medio-alto y el 6 como alto ^18^.

En Colombia, la cobertura en salud se organiza principalmente en dos esquemas: el régimen subsidiado, dirigido a la población pobre o sin capacidad de pago, y los regímenes contributivos, especial y de excepción, para la población con capacidad de pago. El régimen de aseguramiento en salud (RAS) se determinó a partir de la información disponible en el RPCa-M y la consulta en la Base de Datos Única de Afiliados (BDUA), y se clasificó en cinco categorías: i) especial/excepción, ii) contributivo, iii) subsidiado, iv) sin afiliación y v) sin dato. La zona de residencia (ZR) se definió según la ubicación de la vivienda al momento del diagnóstico (urbana o rural). Los casos residentes en zonas rurales (n = 40; 3,1%) se clasificaron como pertenecientes a PSE baja, de acuerdo con las características socioeconómicas predominantes de las veredas donde se registraron.

### Variables del paciente y del tumor

La edad al momento del diagnóstico, la topografía y la morfología se obtuvieron de la base de datos del RPCa-M y se definieron conforme a la Clasificación Internacional de Enfermedades para Oncología, tercera edición, primera revisión (CIE-O 3.1). A los casos con edad desconocida (n = 11) se les imputó la mediana. El puntaje de Gleason y la estadificación se obtuvieron de los reportes de histopatología y de las historias clínicas. Para la estadificación se aplicaron los criterios del sistema Tumor, Nódulo, Metástasis (TNM) esencial propuestos por la Agencia Internacional para la Investigación del Cáncer (IARC) para estudios de base poblacional ^19^. El puntaje de Gleason se categorizó como bajo riesgo (Gleason < 7), riesgo intermedio (Gleason = 7) y alto riesgo (Gleason > 7), de acuerdo con la clasificación de D’Amico ^20^^,^^21^.

### Análisis estadístico

Las variables incluidas fueron de naturaleza cualitativa, medidas en escala nominal u ordinal, y se describieron mediante frecuencias absolutas y relativas. En el análisis descriptivo se incluyeron todos los casos incidentes (N = 1 275); para el análisis de sobrevida se excluyeron los casos captados únicamente por certificado de defunción (DCO, por sus siglas en inglés), debido a que su tiempo de seguimiento es de 0 días y tienden a subestimar la sobrevida ^22^.

El tiempo de sobrevida se calculó como la diferencia entre la fecha de incidencia y la fecha de último contacto, de muerte, de pérdida del seguimiento o de cierre del estudio. La sobrevida a 1, 3 y 5 años se estimó mediante el método de Kaplan-Meier para la cohorte total y de acuerdo con las categorías de RAS, ZR, PSE, grupos de edad (< 50 años, 51-69 años y ≥ 70 años), histología (adenocarcinoma vs. otros), TNM esencial y riesgo según puntaje de Gleason (bajo, intermedio y alto). Las diferencias entre las funciones de sobrevida se evaluaron mediante la prueba de rangos logarítmicos (log-rank).

Se realizaron análisis univariados y multivariados mediante modelos de riesgos proporcionales de Cox. El modelo A incluyó el RAS o la PSE (analizadas por separado), ajustado por edad, histología, estadificación esencial y clasificación de riesgo de Gleason. El modelo B incluyó simultáneamente el RAS y la PSE, además de las variables de ajuste. El supuesto de proporcionalidad de riesgos se verificó mediante los residuos de Schoenfeld. Se evaluaron términos de interacción entre aseguramiento y estadificación, así como entre PSE y estadificación. Debido al bajo número de observaciones en algunas categorías, el RAS se reagrupó en dos categorías: i) contributivo + especial + excepción (población con capacidad de pago) y ii) subsidiado + sin afiliación (población pobre o sin capacidad de pago). La edad se recodificó en dos categorías con punto de corte en 70 años. En los modelos multivariados se excluyeron las categorías “sin dato” o “desconocido” y los casos DCO. El desenlace fue la sobrevida global a 5 años, considerando la muerte por cualquier causa.

El análisis estadístico se realizó con el paquete Stata versión 16.1. En la Figura 1 se presenta el flujograma de selección de los casos.

**Figura 1:** Flujograma de la selección de los casos incidentes con cáncer de próstata (2008-2018) en el Registro Poblacional de Cáncer de Manizales.

### Consideraciones éticas

La investigación fue aprobada por el Comité de Bioética de la Universidad de Caldas mediante el Acta No. 003 del 24 de junio de 2024. Se clasificó como estudio de riesgo mínimo y se ajustó a lo establecido en la Resolución 8430 de 1993 del Ministerio de Salud de Colombia ^23^.

## RESULTADOS

En Manizales se registraron 1 275 casos incidentes de CaP durante el período de estudio. La mediana de edad al momento del diagnóstico fue de 70 años y el promedio de 69,8 años (DE: 9,6). En relación con el régimen de aseguramiento en salud (RAS), el 85,1% de los pacientes pertenecía al régimen contributivo. La posición socioeconómica (PSE) predominante fue la media, con el 47,0% de los casos. El 76,3% residía en la zona urbana al momento del diagnóstico. Se identificaron 26 casos DCO (2,0%). Las características sociodemográficas y tumorales se presentan en la Tabla 1. Los códigos morfológicos registrados en el RPCa-M fueron 8000, 8010, 8140, 8500, 8550, 8574 y 8801, todos con comportamiento maligno. La Figura 2 muestra la distribución por estadificación al diagnóstico. La estadificación según RAS y PSE se presenta en la Figura 3, evidenciando una asociación significativa entre el RAS y la estadificación TNM esencial, con mayor proporción de diagnósticos en estadio localizado limitado entre afiliados a los regímenes contributivo, especial y de excepción, en comparación con los afiliados al régimen subsidiado y no afiliados (59,7% vs. 36,7%; p<0,0001). En el anexo 1 se presenta la distribución de los datos perdidos según el estado del evento.

**Tabla 1:** Características sociodemográficas y clínicas de los pacientes con diagnóstico de cáncer de próstata, según estatus de evento. Manizales, 2008-2018.

		Vivos n (%)	Fallecidos n (%)	Perdidos n (%)	p-valor*
Todos los casos		719 (56,4)	552 (43,3)	4 (0,3)	
Edad					
	< 50 años	17 (2,4)	2 (0,4)	1 (25,0)	p<0,0001
	50 a 69 años	460 (64,0)	149 (27,0)	1 (25,0)	
	70 y más años	242 (33,6)	401 (72,6)	2 (50,0)	
Régimen de Aseguramiento en Salud					
	Especial/excepción	29 (4,0)	17 (3,1)	0 (0,0)	p<0,0001
	Contributivo	630 (87,6)	425 (77,0)	3 (75,0)	
	Subsidiado	48 (6,7)	105 (19,0)	0 (0,0)	
	Sin afiliación	4 (0,6)	1 (0,2)	0 (0,0)	
	Sin dato	8 (1,1)	4 (0,7)	1 (25,0)	
Posición Socioeconómica					
	Alto (estratos 5 y 6)	117 (16,3)	62 (11,2)	1 (25,0)	p=0,050
	Medio (estratos 3 y 4)	333 (46,3)	265 (48,0)	1 (25,0)	
	Bajo (estrato 1, 2 y rural)	111 (15,4)	105 (19,0)	0 (0,0)	
	Sin dato	158 (22,0)	120 (21,8)	2 (50,0)	
Zona de Residencia					
	Urbana	538 (74,8)	418 (75,7)	3 (75,0)	p=0,920
	Rural	24 (3,3)	15 (2,7)	0 (0,0)	
	Sin dato	157 (21,9)	119 (21,6)	1 (25,0)	
Histología					
	Adenocarcinoma	652 (90,7)	386 (69,9)	4 (100,0)	p<0,0001
	Otros** y no especificados	67 (9,3)	166 (30,1)	0 (0,0)	
TNM esencial					
	Localizado limitado	486 (67,6)	236 (42,8)	3 (75,0)	p<0,0001
	Localizado avanzado	47 (6,5)	13 (2,4)	0 (0,0)	
	Metástasis regionales	9 (1,3)	1 (0,1)	0 (0,0)	
	Metástasis a distancia	37 (5,1)	88 (15,9)	0 (0,0)	
	Desconocido	140 (19,5)	214 (38,8)	1 (25,0)	
Riesgo según Gleason					
	Alto	114 (15,9)	111 (20,1)	2 (50,0)	p<0,0001
	Intermedio	209 (29,1)	143 (25,9)	1 (25,0)	
	Bajo	325 (45,2)	116 (21,0)	1 (25,0)	
	Sin dato	71 (9,8)	182 (33,0)	0 (0,0)	
	Periodo de diagnóstico				
	2008-2012	203 (28,2)	268 (48,6)	1 (25,0)	p<0,0001
	2013-2018	516 (71,8)	284 (51,4)	3 (75,0)	

* Prueba exacta de Fisher, excluyendo la categoría "sin dato".

**Sarcoma de células fusiformes y neoplasia maligna

**Figura 2:** Cobertura y frecuencia de la variable Tumor-Nódulo-Metástasis esencial de los casos de cáncer de próstata. Manizales, 2008-2018

**Figura 3:** Distribución de la estadificación Tumor-Nódulo-Metástasis esencial según a) régimen de aseguramiento en salud y b) según posi-ción socioeconómica.

Estadio según aseguramiento en salud: p<0,0001. Estadio según posición socioe-conómica: p=0,16.

La sobrevida global observada a cinco años para el total de la cohorte fue del 76,5%. En la Figura 4 se presentan las curvas de Kaplan-Meier según RAS, PSE, puntaje de Gleason y TNM esencial. En la Tabla 2 se muestran las estimaciones de sobrevida global (SG) a 1, 3 y 5 años por variable. Se observaron diferencias significativas en la SG a cinco años según la edad, con menor sobrevida en los mayores de 70 años. Según el RAS, la SG a cinco años de los pacientes afiliados al régimen subsidiado fue 32 y 27 puntos porcentuales menor que la de los afiliados a los regímenes especial/excepción y contributivo, respectivamente. En cuanto a la PSE, los casos clasificados como PSE baja presentaron una SG a cinco años aproximadamente 11 y 18 puntos porcentuales menor que la observada en la PSE media y alta, respectivamente. No se identificaron diferencias significativas en la SG según la zona de residencia. La histología se asoció de manera significativa con la SG, siendo 30 puntos porcentuales mayor en los pacientes con adenocarcinoma frente a otros tipos histológicos no especificados (81,7% vs. 50,5%). Los pacientes en estadio IV presentaron una SG a cinco años entre 35 y 40 puntos porcentuales inferior a la de aquellos en estadios tempranos. Según el riesgo por Gleason, los pacientes de alto riesgo tuvieron una SG a cinco años 17 puntos porcentuales menor que los de bajo riesgo. No se observaron diferencias significativas por período de diagnóstico.

**Figura 4:** Funciones de Kaplan-Meier según variables del estudio.

**Tabla 2:** Sobrevida global al cáncer de próstata según características sociodemográficas y del tumor. Manizales, 2008-2018.

		N*	Muertes	Sobrevida global			
				12 meses (IC95%)	36 meses (IC95%)	60 meses (IC95%)	**Log-rank** ^**^
Todos los casos		1249	526	92,3 (90,1-93,7)	83,2% (81,0-85,1)	76,5% (74,0-78,8)	
Edad							
	< 50 años	20	2	100% (-)	94,7% (68,1-99,2)	89,5% (64,1-97,3)	p<0,001
	50 a 69 años	609	148	96,6% (94,8-97,7)	91,8% (89,3-93,7)	86,8% (83,9-89,3)	
	70 y más años	620	376	87,9% (85,1-90,2)	74,4% (70,7-77,6)	66,0% (62,1-69,6)	
Aseguramiento en salud							
	Especial/excepción	45	16	97,8 (85,3-99,7)	91,1 (78,0-96,6)	84,4 (70,1-92,3)	p<0,001
	Contributivo	1040	407	93,9% (92,3-95,2)	85,5 (83,2-87,5)	79,4 (76,8-81,7)	
	Subsidiado	146	98	78,1 (70,5-84,0)	63,0 (54,6-70,3)	52,0 (43,6-59,7)	
	Sin afiliación	5	1	100 (-)	100 (-)	100 (-)	
	Sin dato	13	4	100 (-)	91,7 (53,9-98,8)	83,3 (48,2-95,6)	
Posición socioeconómica							
	Alto (estratos 5 y 6)	178	60	98,9 (95,6-99,7)	91,0 (85,8-94,4)	85,4 (79,3-89,8)	p=0,001
	Medio (estratos 3 y 4)	584	250	93,7 (91,4-95,4)	84,4 (81,2-87,1)	78,6 (75,0-81,7)	
	Bajo (estrato 1, 2 y rural)	214	103	87,4 (82,1-91,2)	77,6 (71,4-82,6)	67,7 (61,0-73,6)	
	Sin dato	273	113	89,0 (84,6-92,2)	79,8 (74,5-84,1)	73,1 (67,4-77,9)	
Zona de residencia							
	Urbana	940	399	93,3 (91,5-94,7)	83,8 (81,3-86,0)	77,4 (74,6-80,0)	p=0,495
	Rural	39	15	92,3 (78,0-97,5)	92,3 (78,0-97,5)	79,5 (63,1-89,2)	
	Sin dato	270	112	88,9 (84,4-92,1)	79,6 (74,2-83,9)	72,8 (67,1-77,7)	
Histología							
	Adenocarcinoma	1042	386	95,2 (93,7-96,3)	88,0 (85,8-89,8)	81,7 (79,2-83,9)	p<0,001
	Otros y no especificados	207	140	77,9 (71,6-82,9)	59,1 (52,1-65,5)	50,5 (43,5-57,0)	
TNM esencial							
	Localizado limitado	725	236	96,1 (94,4-97,3)	90,3 (87,9-92,3)	85,2 (82,4-87,6)	p<0,001
	Localizado avanzado	60	13	98,3 (88,6-99,8)	93,3 (83,2-97,4)	88,3 (77,1-94,3)	
	Metástasis regionales	10	1	100 (-)	90,0 (47,3-98,5)	90,0 (47,3-98,5)	
	Metástasis a distancia	121	84	86,0 (78,4-91,0)	65,3 (56,1-73,0)	48,7 (39,5-57,2)	
	Sin dato	333	192	85,0 (80,7-88,4)	72,1 (66,9-76,6)	65,2 (59,8-70,0)	
Riesgo según Gleason							
	Alto	226	110	91,5 (87,0-94,5)	79,5 (73,6-84,2)	71,8 (75,4-77,2)	p<0,001
	Intermedio	353	143	94,1 (91,0-96,1)	87,3 (83,3-90-3)	80,2 (75,6-84,0)	
	Bajo	442	116	98,6 (97,0-99,4)	94,6 (92,0-96,3)	89,3 (86,1-91,9)	
	Sin dato	228	157	78,2 (72,2-83,0)	58,5 (51,9-64,6)	50,7 (44,0-56,9)	
Periodo de diagnóstico							
	2008-2012	458	254	92,6 (89,8-94,7)	83,0 (79,3-86,2)	76,7 (72,5-80,3)	p=0,839
	2013-2018	791	272	92,2 (90,0-93,8)	83,3 (80,5-85,7)	76,4 (73,2-79,2)	

* Se excluyen 26 casos por certificado de defunción.

** Excluyendo las categorías “sin dato”.

En la Tabla 3 se presentan los resultados del análisis multivariado. Se evidenció un efecto significativo del RAS sobre la SG a cinco años: los pacientes afiliados al régimen subsidiado y aquellos sin afiliación -población socialmente más vulnerable- presentaron el doble de riesgo de morir antes de los cinco años del diagnóstico, en comparación con los pacientes con capacidad de pago afiliados a los regímenes contributivo, especial o de excepción, independientemente de la posición socioeconómica, la edad, la histología, la estadificación y el riesgo según el puntaje de Gleason. Los efectos observados para la PSE en el análisis bivariado perdieron significancia estadística en el modelo multivariado tras el ajuste por las demás covariables.

**Tabla 3:** Modelos de riesgos proporcionales de Cox para la sobrevida al cáncer de próstata según aseguramiento en salud y posición socioeconómica. Manizales, 2008-2018.

		Análisis univariado	Análisis multivariado	
		HR (IC 95%)	Modelo A	Modelo B
			HR (IC 95%)	HR (IC 95%)
Aseguramiento				
	Contributivo+Esp./Excep.	Ref.	Ref.	Ref.
	Subsidiado + Sin afiliación	2,67 (2,14 - 3,33)	2,23 (1,60 - 3,11)	2,58 (1,77 - 3,77)
Nivel socioeconómico				
	Alto	Ref.	Ref.	Ref.
	Medio	1,38 (1,04 - 1,83)	1,42 (0,97 - 2,07)	1,30 (0,89 - 1,90)
	Bajo	1,85 (1,35 - 2,55)	1,91 (1,22 - 2,96)	1,47 (0,93 - 2,32)
Edad				
	<70 años	Ref.	Ref.	Ref.
	70 y más años	3,48 (2,87 - 4,21)	2,87 (2,24 - 3,68)	2,65 (2,01 - 3,50)
Subtipo histológico				
	Adenocarcinomas	Ref.	Ref.	Ref.
	Otros y no especificados*	2,96 (2,44 - 3,60)	1,26 (0,71 - 2,20)	1,56 (0,86 - 2,83)
Estadio (TNM esencial)				
	I - Localizado limitado	Ref.	Ref.	Ref.
	II - Localizado avanzado	0,68 (0,39 - 1,19)	0,69 (0,36 - 1,31)	0,88 (0,46 - 1,68)
	III - Metástasis regionales	0,23 (0,03 - 1,65)	0,25 (0,03 - 1,77)	0,47 (0,07 - 3,38)
	IV - Metástasis a distancia	3,60 (2,80 - 4,64)	2,19 (1,61 - 2,98)	2,15 (1,51 - 3,06)
Clasificación de riesgo de Gleason				
	I - Riesgo bajo	Ref.	Ref.	Ref.
	II - Riesgo medio	1,66 (1,30 - 2,12)	1,59 (1,19 - 2,12)	1,34 (0,98 - 1,85)
	III - Riesgo alto	2,41 (1,85 - 3,12)	2,35 (1,73 - 3,19)	2,09 (1,48 - 2,95)

*Otros y no especificados incluyó un caso de sarcoma de células fusiformes y 232 casos con histología no especificada. El modelo A incluye aseguramiento o posición socioeconómica (uno a la vez) ajustado por las demás variables. El modelo A para aseguramiento se ajustó con base en 829 observaciones y arrojó una razón de verosimilitud (Likelihood ratio - LR) = 182,68; el modelo A para nivel socioeconómico se ajustó con base en 652 observaciones y arrojó una LR=116,84. El modelo B se ajustó con un n=649 (número de observaciones con dato conocido para todas las variables incluidas en el modelo), e incluye aseguramiento y posición socioeconómica (simultáneamente), además de las variables de ajuste; el modelo B arrojó una LR=136,13. Se cumplió el supuesto de proporcionalidad, excepto para las categorías “Aseguramiento subsidiado” y “PSE bajo” en el modelo A.

## DISCUSIÓN

Este estudio actualiza las estimaciones de sobrevida de base poblacional en Manizales y evalúa el efecto de variables socioeconómicas, del paciente y del tumor sobre la probabilidad de sobrevida a cinco años posterior al diagnóstico de CaP. En comparación con poblaciones de países desarrollados, la sobrevida observada en Manizales es inferior a la sobrevida relativa (SR) a cinco años de 97,6% reportada por Siegel en Estados Unidos ^24^; no obstante, debe precisarse que las estimaciones de sobrevida global (SG) y SR no son estrictamente comparables debido a las diferencias metodológicas. La sobrevida en Manizales también es inferior al 81,2% reportado en Nueva Zelanda por Matti y Zargar-Shoshtari ^25^, y al 89% informado por Barceló-Obrador a partir de datos del Registro del Cáncer de Mallorca, España ^26^. En el contexto latinoamericano, en México, Torres-Sánchez *et al*., para el período 2012-2016, reportaron una SG a cinco años de 62,0% en pacientes afiliados al Seguro Popular ^27^, mientras que, en Veracruz, Gutiérrez-Juárez *et al*. hallaron una SG de 47,7% para el período 2013-2017 en un hospital de referencia regional ^11^.

En Colombia, la sobrevida en Manizales es inferior a la reportada en Cali, pero superior a la registrada en Bogotá. Bravo et al. analizaron el período 1998-2017 en el Registro Poblacional de Cáncer de Cali y documentaron una sobrevida neta (SN) variable según el período: 79,9% (1998-2002), 90,2% (2003-2007), 87,5% (2008-2012) y 90,1% (2013-2017) ^28^. Aunque estas estimaciones corresponden a SN y no a SG, ofrecen un marco de referencia nacional. En contraste, en un estudio hospitalario en Bogotá, Campos-Guzmán reportó una SG a cinco años de 57,0% para el período 2008-2014 ^7^. Comparada con el estudio previo en Manizales, la SG a cinco años observada en esta cohorte muestra una mejora de cinco puntos porcentuales frente al 71,1% reportado por Arias-Ortiz y De Vries para 2003-2007 ^9^.

Como era esperable, la edad mayor de 70 años se asoció con un riesgo aproximadamente dos veces mayor de morir antes de los cinco años posteriores al diagnóstico. De acuerdo con la IARC, la incidencia de CaP en personas mayores de 65 años aumentó sostenidamente entre 1988 y 2007, y se proyecta que hacia 2030 los países en vía de desarrollo tendrán una incidencia ajustada por edad mayor que los países desarrollados ^29^. El mayor riesgo de muerte en pacientes de mayor edad es consistente con lo descrito por Bernard et al. en Estados Unidos, quienes reportaron que el diagnóstico a los 75 años o más constituye un factor de riesgo significativo para mortalidad por CaP (HR: 1,5; IC 95%: 1,4-1,6) ^4^, así como con estudios hospitalarios previos en Colombia ^7^^,^^8^.

En cuanto a la posición socioeconómica (PSE), el análisis bivariado mostró una asociación directa con la sobrevida, evidenciando menor SG a medida que disminuye la PSE. En población estadounidense se ha demostrado mayor riesgo de muerte por CaP en hombres con menor nivel educativo y que residen en barrios de menores ingresos, incluso en aquellos con alto nivel educativo pero que viven en entornos desfavorecidos (HR: 1,4; IC 95%: 1,1-1,8) ^30^. De manera similar, el Registro Nacional de Cáncer de Suecia encontró que los pacientes con menor estatus socioeconómico eran diagnosticados en estadios más avanzados, y que la mortalidad por todas las causas fue significativamente mayor en el cuartil de menores ingresos en comparación con el de mayores ingresos (30% vs. 12%), con mayores tasas de curación en los grupos más favorecidos (OR: 1,8; IC 95%: 1,6-1,9) ^31^. En México, Gutiérrez-Juárez *et al.* reportaron que a mayor grado de marginalidad existía mayor probabilidad de morir antes de cinco años (HR: 2,3; IC 95%: 1,5-3,7) ^11^. En Costa Rica, pese a contar con un sistema de salud universal, también se han documentado inequidades sociales en la sobrevida al cáncer ^32^. En Colombia, Restrepo et al. hallaron mayor sobrevida en personas con PSE media y alta frente a PSE baja en Cali ^10^.

Respecto al régimen de aseguramiento en salud (RAS), este estudio confirma que pertenecer al régimen subsidiado o no estar afiliado es un predictor independiente de menor sobrevida en comparación con estar afiliado a los regímenes contributivo, especial o de excepción. En Estados Unidos, Krimphove *et al.* encontraron que los pacientes afroamericanos con CaP avanzado presentan mayor riesgo de mortalidad asociado, entre otros factores, a una menor cobertura por seguros privados ^33^. De igual forma, Myint *et al*. reportaron diferencias significativas en la sobrevida a cinco años entre pacientes sin seguro o con seguros públicos frente a pacientes con seguro privado (HR entre 1,3 y 2,1) ^34^. También se ha documentado que el aseguramiento público o la ausencia de seguro se asocian con menor acceso al tratamiento del CaP ^35^. En Manizales, las diferencias de sobrevida según RAS podrían explicarse, al menos parcialmente, por barreras en el acceso al diagnóstico temprano, dado que la proporción de estadios iniciales fue menor entre pacientes del régimen subsidiado y no afiliados ^9^. A pesar de que la legislación colombiana garantiza la atención oncológica independientemente del tipo de aseguramiento ^36^, los resultados evidencian que persisten brechas de inequidad que requieren intervenciones más efectivas.

No se encontraron diferencias significativas en la sobrevida según zona de residencia, en concordancia con el estudio previo de Manizales ^9^. Este hallazgo contrasta con lo descrito en Veracruz, México, donde los pacientes rurales presentaron estadios más avanzados al diagnóstico y menor sobrevida (HR: 1,7; IC 95%: 1,2-2,4) ^11^. En Manizales, este resultado podría explicarse por la baja proporción de población rural (6,3%), lo que limita la potencia estadística para detectar diferencias ^37^.

En relación con las características tumorales, la frecuencia de estadio localizado limitado (59,6%) fue inferior a la suma de los estadios I y II reportados en la revisión nacional de Solano-Dazzarola *et al*. ^38^. Las diferencias de sobrevida por estadificación observadas fueron consistentes con lo descrito en la literatura ^38^. Asimismo, los resultados según el puntaje de Gleason concuerdan con estudios hospitalarios en Colombia ^7^^,^^8^^)^ y con investigaciones de México ^27^ y España ^26^^,^^39^.

Los modelos de Cox utilizados se fundamentaron en el marco teórico de las inequidades en salud y en evidencia previa sobre el efecto de la posición socioeconómica, la ruralidad y el aseguramiento en salud sobre la sobrevida oncológica ^(^^35^^,^^40^^-^^44^.

Entre las principales limitaciones del estudio se encuentra el porcentaje de datos faltantes en histología, estadificación y puntaje de Gleason, lo cual pudo haber introducido sesgo de información, particularmente por la mayor proporción de datos perdidos entre los fallecidos. Adicionalmente, el uso del estrato socioeconómico del barrio como variable sustituta de la PSE individual podría generar confusión residual. Otra limitación relevante es la ausencia de información sobre tratamientos recibidos. Asimismo, al no disponerse de causas específicas de muerte, se estimó únicamente la sobrevida observada, lo que limita la interpretación causal; en futuros estudios sería deseable aplicar métodos de sobrevida neta ^45^. Tampoco se contó con información sobre etnicidad, nivel educativo ni adherencia terapéutica, variables relevantes en la sobrevida al CaP.

Como fortalezas, este es un estudio de base poblacional con diez años de seguimiento, sustentado en un registro que cumple estándares internacionales, lo que favorece la comparabilidad de los resultados. Su naturaleza poblacional permite reflejar condiciones reales de la atención en salud. El bajo porcentaje de pérdidas en el seguimiento (<0,5%) fortalece la validez de las estimaciones.

En futuras investigaciones será clave incorporar variables adicionales como niveles de PSA, indicadores individuales de nivel socioeconómico, datos detallados de tratamiento, adherencia terapéutica y causas específicas de muerte. Además, estudios con enfoque cualitativo permitirán profundizar en la percepción de la enfermedad, el acceso a la atención y la calidad de vida.

En conclusión, la sobrevida global al CaP en Manizales durante el período 2008-2018 continúa siendo inferior a la observada en países desarrollados. Aproximadamente entre siete y ocho de cada diez pacientes sobreviven cinco años después del diagnóstico. Persisten brechas significativas de sobrevida según el régimen de aseguramiento en salud, en desventaja de la población socialmente vulnerable, posiblemente mediadas por diagnósticos tardíos y barreras de acceso al tratamiento, independientemente de la posición socioeconómica, la edad, la histología, la estadificación y el riesgo según el puntaje de Gleason.
